# Cholesterol, C-Reactive Protein, and Periodontitis: HMG-CoA-Reductase Inhibitors (Statins) as Effect Modifiers

**DOI:** 10.5402/2011/125168

**Published:** 2011-11-16

**Authors:** Peter Meisel, Thomas Kohlmann, Henri Wallaschofski, Heyo K. Kroemer, Thomas Kocher

**Affiliations:** ^1^Department of Pharmacology, University of Greifswald, 17475 Greifswald, Germany; ^2^Department of Dental Clinics, Unit of Periodontology, University of Greifswald, 17475 Greifswald, Germany; ^3^Department of Community Medicine, University of Greifswald, 17475 Greifswald, Germany; ^4^Department of Clinical Chemistry, University of Greifswald, 17475 Greifswald, Germany

## Abstract

Common risk factors of periodontitis and cardiovascular diseases fuel the debate on interrelationships between them. The aim is to prove whether statins may influence periodontal parameters by affecting either of these factors. Out of the 4,290 subjects of SHIP (Study of Health in Pomerania), we included subjects aged >30 years (219 with statins, 2937 without) and excluded edentulous. We determined periodontal measures, cholesterol fractions, and inflammation markers. Statin use and periodontal risk factors were assessed. Gingival plaque and periodontal attachment loss were associated with systemic LDL cholesterol (*P* < 0.001) and C-reactive protein CRP (*P* = 0.019) revealing interaction with statin use. When adjusted for age, sex, smoking, diabetes, education, and dental service, statins were identified as effect modifiers abolishing the relationship between attachment loss and LDL and between gingival plaque and LDL (interactions *P* < 0.001). No statin-related interaction was detected with increase in CRP. The interaction supports the view of inter-relationships between periodontal and systemic inflammatory mediators.

## 1. Introduction

Periodontitis is an inflammatory disease caused by periodontopathogenic bacteria residing in dental plaque, that is the biofilm on the tooth surface. It is accompanied by pocket formation and leads to attachment loss and eventually results in tooth loss. It affects a majority of all adults and is consequently a severe medical challenge.

In recent years, various studies revealed that the local inflammation within the oral cavity is associated with increased levels of markers of systemic diseases. Special attention was given to the relationships between periodontal diseases and diabetes, cardiovascular disease, and osteoporosis, among others [1]. Increased blood levels of HbA_ 1c_, CRP, and cytokines supported the idea that periodontitis may affect or worsen systemic diseases. However, there is much dispute about the direction of cause and effect [2, 3]. A bidirectional relationship may exist between the local periodontitis and systemic diseases, both of which are inflammatory in nature. Among the factors for which an association with the severity of periodontitis was reported are the phospholipids, cholesterol, and its subfractions LDL and HDL (LDL-c, HDL-c). This may be of special interest as it was hypothesized that there is a close inter-relationship between cardiovascular diseases and periodontitis. These conditions share common risk factors such as smoking, diabetes, and low socioeconomic status.

There is substantial evidence that periodontal diseases are associated with elevated systemic cholesterol concentrations. Presumably, the association between periodontitis and lipid levels is the consequence of systemic effects of inflammatory stimuli [4]. Concentrations of C-reactive protein (CRP) are elevated in both conditions.

The HMG CoA reductase inhibitors or statins are the most effective agents currently available to lower LDL cholesterol levels effectively. Several clinical trials have demonstrated a considerable reduction of cardiovascular risk. In addition, recent studies suggest that the statins may have other effects beyond the lipid reducing action, especially with respect to inflammatory events [5, 6]. Thus, there was some interest to study statin effects in periodontitis [7, 8]. It was shown that patients on statin medication exhibited reduced periodontal injury as compared to subjects without the drugs [9]. Statin medication may have some beneficial effects when subjects have dental plaque or signs of periodontitis as gingival bleeding [10]. Thus, the question arises whether statins may have an influence on periodontal parameters by affecting either the inflammation or the association between the disease and the cholesterol concentration or both.

Hitherto, it is unclear if the association is caused by the lipids or the inflammation. Perhaps there is a susceptible phenotype prone for both, increased lipid levels and inflammation. With these associations, it seems reasonable to hypothesize that statins could also have an influence on the extent or severity of periodontitis by affecting lipid levels and/or inflammatory stimuli. Thus, it is the aim of this study to elucidate the statin effects on periodontal disease measures. This study elucidates such effects in a population-based health survey study.

## 2. Participants and Methods

### 2.1. Study Population

7,008 subjects were selected by a cluster sampling method from a population of 210,000 inhabitants of the northeastern German region of Pomerania designated as SHIP study (Study of Health in Pomerania). From 32 communities in the region, a random sample was drawn from residence registries, stratified by gender and age. Finally, 7,008 subjects were sampled, with 292 persons of each gender in each of twelve five-year age strata (age range 20–80 years). The net sample (without migrated or deceased persons) consisted of 6,267 eligible subjects. The final SHIP sample comprised 4,310 participants (response 68.5% of eligible subjects), of which 4,290 subjects underwent dental examinations. The design of the SHIP study, recruiting of participants, and the scope of this population-based cross-sectional health survey was reported elsewhere [11]. The participants gave their written informed consent and the study was approved by the local ethics committee. 

### 2.2. Anamnestic and Periodontal Examinations

Periodontal status was assessed by specially trained dentists (intraclass correlation of examiners 0.82–0.91). The examination was conducted under clinical conditions in a dental chair, participants supine, and under optimal lighting, but without saliva ejector or air drying. Assessment included probing depth, clinical attachment loss (CAL), plaque, bleeding on probing, and the number of teeth. Because of the efficiency afforded, the periodontal examination was carried out according to the half-mouth method on the left or right side in alternate subjects. All fully erupted teeth were assessed excluding third molars. A maximum of 14 teeth per subject was examined. Attachment loss and probing depth were assessed with a periodontal probe (PCP 11, HuFriedy, Chicago, IL) at mesiobuccal, distobuccal, midbuccal, and midlingual aspect on each selected tooth. Attachment loss is represented by the distance from the cementoenamel junction to the bottom and probing depth by the distance from the gingival margin to the bottom of the periodontal pocket. The measurements were made in whole millimeters.

Visual inspection and probing determined the presence or absence of marginal plaque. Assessment was performed at four sites per tooth as mentioned above at three teeth (midincisor, canine, and first molar) in ipsilateral quadrants (1 and 4 or 2 and 3 alternating from one patient to the next) using a dental probe. If a test tooth was missing, the distally adjacent tooth was examined instead. Results were expressed as percentage of sites with plaque.

Smoking behaviour and socioeconomic status were assessed with an extensive questionnaire and an interview. Likewise, the use of medications was assessed and recorded according to the ATC index [12]. 280 participants were treated with the following drugs: simvastatin (*n* = 87), lovastatin (*n* = 27), pravastatin (*n* = 53), fluvastatin (*n* = 37), atorvastatin (*n* = 34), and cerivastatin (*n* = 42). 123 participants were additionally treated with *β*-blockers and 74 with calcium antagonists. Fibrates were used by 55 other participants. In order to confirm a regular intake of the statins, subjects were asked to present evidence of their use, that is prescriptions or package inserts. Of the 280 subjects treated with statins remained 219 in the final analysis after exclusion of the edentulous from which no periodontal parameters were available.

### 2.3. Clinical Chemistry and Statistics

Total cholesterol, LDL-c, HDL-c, fibrinogen according to the Clauss method, and HbA_ 1c_ were determined by standard laboratory methods. High-sensitivity C-reactive protein (CRP) was determined in serum by particle-enhanced immunonephelometry (hsCRP kit, Dade Behring Inc.) with a test sensitivity of 0.2 mg/L. Mann-Whitney or Kruskal-Wallis tests were used to compare the means of two or three groups, respectively. Periodontal parameters plaque and clinical attachment loss (CAL) were divided into tertils for stratification, severe cases defined with a mean CAL > 4 mm. In logistic regression analyses, mean CAL or plaque were used as continuous variables. To improve the precision and to reduce correlation between variables, mean CAL and plaque were centered by subtracting the overall mean value from each observed value [13]. Clinically relevant cutoff levels of LDL cholesterol (LDL-c) with respect to cardiovascular risks were used: <100 mg/dL (2.58 mmol/L, optimal), 100 to 160 mg/dL (above optimal or borderline), and >160 mg/dL (4.14 mmol/L, high). For CRP levels an arbitrary threshold of 2 mg/L was set [14]. Kruskal-Wallis test was used for comparisons, logistic regression for multivariate analyses. The statistical software STATVIEW 5.0 (SAS, Cary, NC) was employed.

## 3. Results

Among the 4,310 participants of the SHIP, we identified 280 subjects who were treated with statins. Most of them were in their later life period. Age is an important confounder for all variables studied. The prevalence of periodontitis increases with increasing age as it is true for the concentration of LDL cholesterol. The frequency of statin medications increases with age as well. For this study, a subpopulation was selected with the participants who were aged 30 and older (in younger participants there were no statin users) excluding all edentulous subjects. 3,156 participants were thus included in this analysis. The characteristics of these subjects are given in Table 1 along with their LDL-c status. Subjects with high LDL-c levels were older than subjects within the optimal concentration range. Correspondingly, periodontal parameters were significantly higher as it was true for markers of systemic inflammation (CRP, fibrinogen) or of glucose homeostasis (HbA_ 1c_). Frequency of statin use was only a third of subjects in the high LDL-c group as compared with the optimal LDL-c group. We must be aware that there are presumably four different categories of participants, namely individuals (i) with low LDL-c and without the drug, (ii) with low LDL-c receiving the drug, either for prophylaxis or after successful decrease of the lipid, (iii) not taking statins despite elevated LDL-c levels, and (iv) persons who take the drug but their LDL-c is still above the clinically relevant cutoff level.

**Figure 1:**
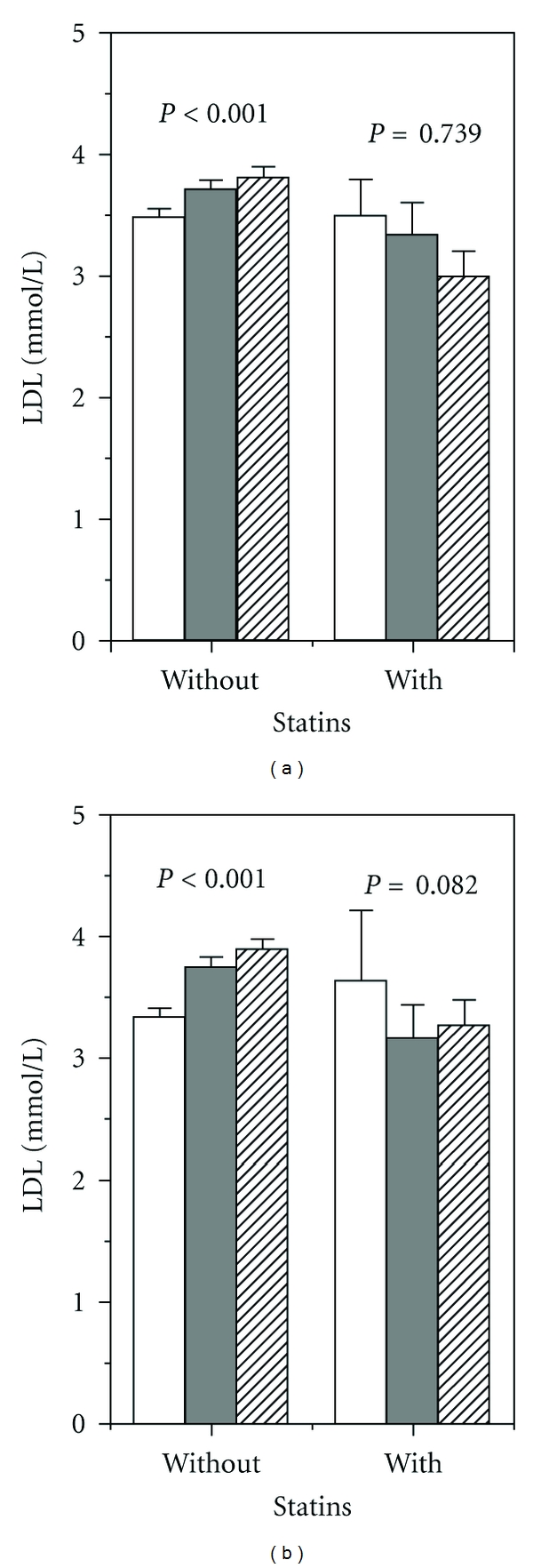
(a) Mean LDL-c concentration related to increasing extent of gingival plaque (in tertils) without and with statin use. ANOVA: statins *P* < 0.001, plaque tertils *P* = 0.475, interaction *P* < 0.001. (b) Mean LDL-c dependent on severity of attachment loss (mean CAL tertils) without and with statin use. ANOVA: statins *P* = 0.003, CAL tertils *P* = 0.366, interaction statins ∗CAL *P* = 0.002. Lowest periodontal tertil—white columns, 2nd tertil—dark columns, 3rd tertil—hatched columns.

**Figure 2:**
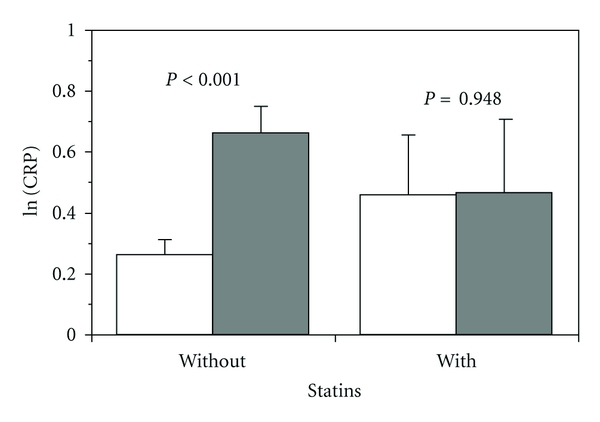
Severe periodontitis (mean CAL > 4 mm, dark columns; white columns CAL ≤ 4 mm) is associated with an increase in CRP, which is attenuated by statins. ANOVA: statins *P* = 0.992, CAL *P* = 0.019, interaction *P* = 0.024.

A first crude stratification according to the extent of gingival plaque (Figure 1(a)) or the severity of attachment loss (Figure 1(b)) resulted in the plots showing the level of LDL-c with and without statins with increasing periodontitis. In each case, the differences associated with statin use suggest the presence of interaction. The statins themselves or the medical indications for their use affect the relationship between the dental parameters and their systemic correlate in such a way that the increased LDL-c associated with higher plaque or attachment loss was attenuated. Similarly, CRP levels were increased in subjects with periodontitis, an increase which was not observed in subjects under statin medication (Figure 2).

We performed multiple regression analyses with the borderline and the high LDL-c levels as dependent variables, adjusting for potential confounding or influential factors. Periodontal parameters were associated with increased risk of high LDL-c. Statin use showed lower risk exhibiting significant interaction with the periodontitis factors. A comparison of the odds ratios between medium and high LDL as dependent variables revealed a reasoned gradation for the periodontitis parameters and the interaction factors with statins (optimal <100 mg/dL as reference). Statin interaction with plaque (Table 2) resulted in odds ratio OR = 0.96 and 0.77 (*P* < 0.001) for LDL-c 100–160 mg/dL and >160 mg/dL, respectively. Interaction figures for mean attachment loss in mm (Table 3) were OR = 0.98 (*P* = 0.046) and 0.96 (*P* < 0.001), respectively.

If no interaction exists, then the increased risk of having high LDL-c levels due to periodontitis would be the same regardless of the individual's drug treatment. Since statin use and gingival plaque or attachment loss interact, the risk of high LDL-c from an increase in periodontitis figures is associated with the drug. Probably, statins reduce LDL-c so effectively that it remains low irrespective of whether there is plaque-related challenge or not. Likewise, since statin use and attachment loss interact in this analysis, the risk of high LDL-c concentrations from an increase in attachment loss may be modulated by the drug. In this sense statins act as effect modifiers.

Similarly, we performed logistic regression analyses with a CRP cutoff of 2 mg/L as dependent variable and identical independent variables as in Table 2. Odds ratios of belonging to the high CRP group OR = 1.06 (1.04–1.09, *P* < 0.001) and OR = 1.11 (1.05–1.17, *P* < 0.001) were calculated for plaque (10% increments) and mean attachment loss in mm, respectively. The probability of belonging to the high CRP group was insignificantly lower with statins (OR = 0.79, C.I. 0.58–1.07, *P* = 0.123). There was no interaction with these periodontal measures. 

In order to address the question if the subjects treated with statins were different from those without, we compared different risk factors among these groups. Subjects treated with statins or not were basically different from each other, especially those with elevated systemic inflammation marker CRP (data not shown). The subjects without statins had lower socioeconomic status as indicated by education and income. Signs of poor oral hygiene were indicated by high extent of plaque and bleeding on probing. Thus, different reasons for prescribing statins may contribute to the relationships shown.

## 4. Discussion

In this study we reported that the statistical association of gingivitis and periodontitis with risk factors for cardiovascular sequelae was modulated by statins. After stratification according to periodontal measures, an effect modification by statins affecting the LDL-c level was observed. 

Gingival plaque is a biofilm which consists of numerous different bacterial species nourished from bleeding gingiva, saliva, and crevicular fluid. In general, it is believed that this biofilm induces an inflammatory response to counteract the bacterial challenge. On the other hand, gingival plaque accumulates preferentially on those sites which are deteriorated by inflammation [15]. Various reports have demonstrated an effect of dental plaque on the systemic inflammatory response [16] and on the level of circulating lipids including cholesterol [17]. This mutual influence of inflammation and bacterial biofilm has its mirror image in the relation between periodontal inflammation and systemic reactions as reflected by increases in phospholipids, cytokines, and markers of inflammation, namely, fibrinogen, CRP, and so forth, which may potentially mediate the connection between periodontal and cardiovascular diseases [18].

Intervention studies reporting effects of periodontal treatment on systemic parameters such as cholesterol and CRP give evidence of a causal direction oral inflammation  > systemic effect. Chronic inflammation induced by plaque leads to attachment loss and finally to tooth loss. As suggested by the associations shown, there are different inter-relationships between the local inflammation and the systemic responses. Periodontitis and cardiovascular diseases share common risk factors, and several reports showed that periodontitis is accompanied by systemic inflammation and increasing concentrations of circulating phospholipids [18, 19] as well as markers of inflammation [20]. Plaque removal by local periodontal therapy reduces total and LDL-c as well as circulating CRP [21]. The association shown in our study regarding the statin effects may lead to speculations that also an opposite relationship could exist. Notwithstanding, a quite simple explanation for the effects shown is that the statins affect LDL-c and CRP in a way preventing any increase from periodontal stimuli. 

With regard to odontogenic septicemia it was speculated whether statins could play a role in the interaction between periodontitis and cardiovascular diseases [22]. However, antimicrobial effects of statins on dental biofilm bacteria as claimed by some authors [23] are less probable as statins are much less potent inhibitors of bacterial HMG-CoA reductases than of the human enzyme variant [24]. Elevated levels of CRP are associated with infection by subgingival organisms related to periodontal disease, including *Porphyromonas gingivalis *and *Prevotella intermedia* [25]. It has been shown that high glucose concentrations enhance LPS-induced expression of inflammatory cytokines and simvastatin largely blocks this enhanced expression [26]. 

In vitro experiments in cell lines modelling gingival tissue revealed that simvastatin reduces the interleukin-1*α*-induced production of inflammatory cytokines IL-6 and IL-8 [23]. This effect resulted from specific inhibition of HMG-CoA reductase. Thus, the association shown here reflect probably diminished LDL-c levels in the systemic circulation associated with reduced inflammation in the gingiva and concomitantly reduced plaque. On the other hand, complete extraction of all teeth (and thus, removal of all plaque) reduces the circulating markers of inflammation, namely, CRP, fibrinogen, and white blood cells [27]. A significantly reduced number of pathological periodontal pockets in patients taking simvastatin or atorvastatin has been reported [9]. Moreover, treatment of periodontitis is followed by a decrease in systemic markers of inflammation including IL-6 [28, 29], an effect mimicked in vitro by simvastatin in epithelial cells [22]. Statins also reduce the production of IL-1*β* and TNF*α*, inflammatory cytokines which are known to play a pivotal role in periodontitis [30]. While the LDL-c reduction by statins has unequivocally beneficial consequences, the effects induced by lowering the inflammatory markers are less clear [31]. Most of the widely discussed “pleiotropic” effects of statins correlate with the extent of lipid lowering, for example, the reduction of CRP [29]. 

The study has some limitations. There was no information available on how long statins were already used, although these drugs are supposed to be taken for chronic use. Different types of statins were lumped together. Notwithstanding, all statins have shown similar efficacy in reducing CRP levels [32]. Due to the cross-sectional setting, a causative sequence cannot be deduced from the interactions of cholesterol concentrations, CRP levels, periodontal measures, and statin treatment. The sequence of effects could not be elucidated. 

In this study, we described the statins as effect modifiers in the relationship between periodontitis and its inflammatory counterparts in the systemic circulation. Statins could attenuate the inflammation-related systemic effects. Controlled clinical trials using systemically or locally administered statins have shown significant improvements of periodontal sequelae [33, 34].

## 5. Conclusion

Gingival plaque and periodontal attachment loss were associated with elevated levels of systemic LDL/HDL cholesterol and C-reactive protein. In participants treated by statins, this periodontitis-related increase was abolished. If confirmed by clinical studies, the lipid-lowering statins would be promising drugs to prevent periodontitis-related increases in cardiovascular risk markers.

## Figures and Tables

**Table 1:** Characteristics of the subjects and their distribution according to their serum LDL cholesterol state.

	LDL concentration range, mg/dL			
Subjects with	**<**100	**≥**100–<160	**≥**160	*P*
Number of subjects	545	1679	942	—
Age, years	47.9 ± 13.3	51.7 ± 13.4	54.5 ± 12.1	<0.001
Dentist seen >1 year before*	9.4	10.4	11.8	0.309
Education <10th grade*	31.0	37.9	46.5	<0.001
Smoking, pack years	8.4 ± 13.7	8.7 ± 14.2	9.9 ± 15.1	0.882
Alcohol, g/weekend	47 ± 65	45 ± 59	41 ± 53	0.042
Statin medication*	11.6	7.1	3.9	<0.001

*Metabolic characteristics*				
LDL, mmol/L	2.1 ± 0.4	3.4 ± 0.4	5.0 ± 0.8	<0.001
HDL, mmol/L	1.6 ± 0.6	1.4 ± 0.4	1.4 ± 0.4	<0.001
hsCRP, mg/L	2.9 ± 5.1	2.8 ± 4.7	3.0 ± 6.8	0.003
Fibrinogen, g/L	2.9 ± 0.6	3.0 ± 0.7	3.2 ± 0.7	<0.001
HbA_1c_, %	5.3 ± 0.9	5.4 ± 0.9	5.6 ± 0.9	<0.001

*Periodontal characteristics*				
No. of teeth^†^	19.9 ± 7.2	19.0 ± 7.7	17.6 ± 8.0	<0.001
Attachment loss, mean mm	2.5 ± 1.8	2.9 ± 1.9	3.3 ± 1.9	<0.001
Probing depth, mean mm	2.5 ± 0.7	2.6 ± 0.8	2.7 ± 0.8	<0.001
**Attachment loss, % ≥4 mm^‡^**	10 (40)	21 (52)	31 (60)	<0.001
**Plaque, % of sites^‡^**	50 (54)	54 (51)	60 (54)	<0.001
BOP, % of sites^‡^	29 (38)	35 (42)	38 (44)	<0.001

*Percent of subjects, ^†^excluding the 3rd molars and zero figures, ^‡^median (interquartile range).

**Table 2:** Logistic regression: dental plaque (extent in 10% increments) and the risk of belonging to the LDL concentration range 100–160 mg/dL or >160 mg/dL with reference LDL < 100 mg/dL.

Dependent variable	LDL 100–<160 mg/dL		LDL ≥ 160 mg/dL	
	OR (95% CI)	*P*	OR (95% CI)	*P*
Plaque, crude	1.03 (1.00–1.06)	0.099	1.08 (1.04–1.12)	<0.001
Model 1*				
Plaque	1.00 (1.00–1.04)	0.998	1.03 (0.99–1.07)	0.130
Model 2*				
Plaque	1.00 (0.97–1.04)	0.990	1.04 (1.00–1.08)	0.081
Statins	0.37 (0.26–0.54)	<0.001	0.14 (0.09–0.23)	<0.001
Plaque × statin use	0.96 (0.86–1.08)	0.512	0.77 (0.66–0.89)	<0.001

*Model 1: adjusted for age (continuous), sex (male 0, female 1), HbA_1c_ (continuous), education (10th grade or higher), smoking (pack years), alcohol consumption (g/weekend), and last dental visit (within the last year 0, longer than one year 1).

*Model 2: as model 1 with the additional variables shown.

**Table 3:** Logistic regression: mean clinical attachment loss (CAL) and the risk of belonging to the LDL concentration range 100–160 mg/dL or >160 mg/dL with reference LDL < 100 mg/dL.

Dependent variable	LDL 100–<160 mg/dL		LDL ≥ 160 mg/Dl	
	OR (95% CI)	*P*	OR (95% CI)	*P*
CAL, mm, crude	1.17 (1.10–1.25)	<0.001	1.30 (1.21–1.39)	<0.001
Model 1*				
CAL mean, mm	1.08 (1.00–1.16)	0.064	1.11 (1.02–1.21)	0.013
Model 2*				
CAL mean, mm	1.11 (1.02–1.20)	0.020	1.21 (1.10–1.33)	<0.001
Statins	0.36 (0.24–0.53)	<0.001	0.14 (0.08–0.22)	<0.001
CAL × statin use	0.98 (0.97–1.00)	0.046	0.96 (0.94–0.98)	<0.001

*Model 1: adjusted for age (continuous), sex (male 0, female 1), HbA_1c_ (continuous), education (10th grade or higher), smoking (pack years), alcohol consumption (g/weekend), and last dental visit (within the last year 0, longer than one year 1).

*Model 2: as model 1 with the additional variables shown.
